# Uncovering and Autonomous Eruption of Palatally Impacted Canines—A Case Report

**DOI:** 10.3390/dj9060066

**Published:** 2021-06-09

**Authors:** Alessandra Impellizzeri, Martina Horodynski, Emanuela Serritella, Gaspare Palaia, Adriana De Stefano, Antonella Polimeni, Gabriella Galluccio

**Affiliations:** Department of Oral and Maxillofacial Sciences, “Sapienza” University of Rome, Via Caserta 6, 00161 Roma, Italy; alessandra.impellizzeri@uniroma1.it (A.I.); martinahorodynski@gmail.com (M.H.); emanuela.serritella@uniroma1.it (E.S.); aadestefano@hotmail.com (A.D.S.); antonella.polimeni@uniroma1.it (A.P.); gabriella.galluccio@uniroma1.it (G.G.)

**Keywords:** laser CO_2_, ortho-surgical disinclusion, palatally impacted canines, no orthodontic traction, case report

## Abstract

The impaction of permanent maxillary canine is a common clinical occurrence, and it is observed in 2% of patients who require orthodontic treatment. This case report describes a new orthodontic-surgical approach through the use of CO_2_ laser, for the exposure of the palatally impacted canines. A 13-year-old female referred to our observation to make an orthodontic examination because of the maxillary primary canines’ persistence in upper arch. Orthopanoramic X-ray showed impaction of both permanent maxillary canines. The family history revealed that the patient’s mother had the same orthodontic problem. Cone Beam Computer Tomography (CBCT) was requested to plan the surgical-orthodontic treatment. Surgical exposure of the impacted canines was performed using a CO_2_ laser and subsequent periodontal pack application. No orthodontic devices were applied for impacted teeth traction on dental arch. Canines’ movement was monitored at 1, 8 and 16 weeks post-surgery with photo and intraoral scanner CS3500 (CS3500^®^, Carestream Health, Atlanta, GA, USA). When canine crowns were completely erupted on palatal side, the alignment in the arch with indirect bonding technique was performed. Complete disimpaction of canine crowns was obtained in only four months. As reported in the literature, this case confirms that impacted canines’ exposure to CO_2_ laser has advantages if compared with traditional surgery: no bleeding during and after the procedure, decontaminant effect on the surgical area, no suture, and a fast spontaneous eruption. Conclusions: The pre-orthodontic uncovering and autonomous eruption of palatally impacted maxillary canines provides simplified, predictable, and more aesthetic outcomes. Furthermore, a significant positive factor is that there is no need to carry out the orthodontic traction of the impacted element, undoubtedly better compliance by the patient during the next alignment phase with the fix orthodontic appliance.

## 1. Background

The impaction of a permanent maxillary canine is a common clinical occurrence, and it is observed in 2% of patients who require orthodontic treatment. The upper canine is one of the teeth that mostly undergoes impaction, second only to third molars, with a prevalence range ranging from 0.8% to 5.2% depending on the population examined. In Italy, it settles around 2.4%. The incidence of maxillary canine impaction is more than double that in the jaw, and the ratio of palatal inclusion to buccal one is 8 to 1. Of all patients who have impacted maxillary canines, 8% have bilateral impactions, and they are twice more common in females than males [1]. The cause of impacted maxillary canines is not exactly known; it is commonly thought that palatally impacted canines are associated with hypoplastic or missing lateral incisors (“the guidance theory”) or with aplasia of premolars and third molars and supernumerary teeth (“the genetic theory”).

One of the fundamental aspects in the diagnosis and treatment planning of a complex tooth anomaly such as the impaction of the permanent maxillary canine is the ability to recognize the tooth displacement early and to predict the subsequent failure of eruption. The early diagnosis of canine displacement in relation to the surrounding structures is based primarily on radiographic examination. Erikson and Kurol found that the more mesially located the crown of the canine in the panoramic film, the more reduced the likelihood of canine eruption [1,2].

Orthodontic treatment of the impacted upper canine is still considered a difficult challenge for clinicians, and it requires a multidisciplinary therapeutic approach. The therapeutic approach involves surgical exposure of the included tooth, followed by orthodontic traction to guide and align the tooth to the dental arch. For palatally impacted canines, various surgical approaches and various orthodontic traction techniques have been described in the literature [3,4,5,6].

This case report proposed a new orthodontic surgical approach through the use of CO2 laser, for the disinclusion of the palatally impacted canines. Furthermore, the literature reports that high-intensity laser therapy (HILT), “surgical laser”, having a cutting action on the soft tissues [7,8], can also be used for opercolectomy, removing the soft tissue that overlies the impacted tooth.

## 2. Case Presentation

**Figure 1:**
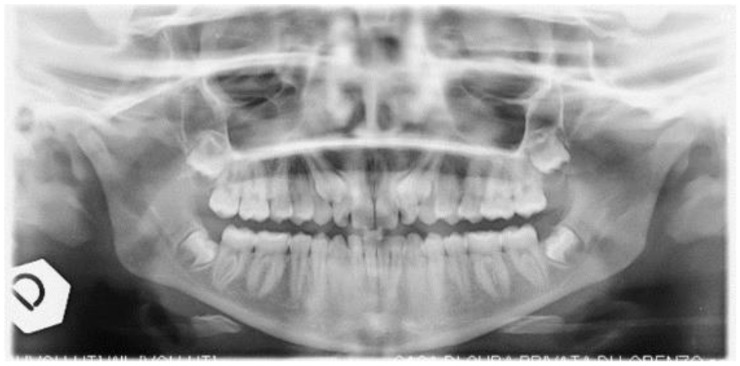
Orthopanoramic X-ray pre-treatment.

**Figure 2:**
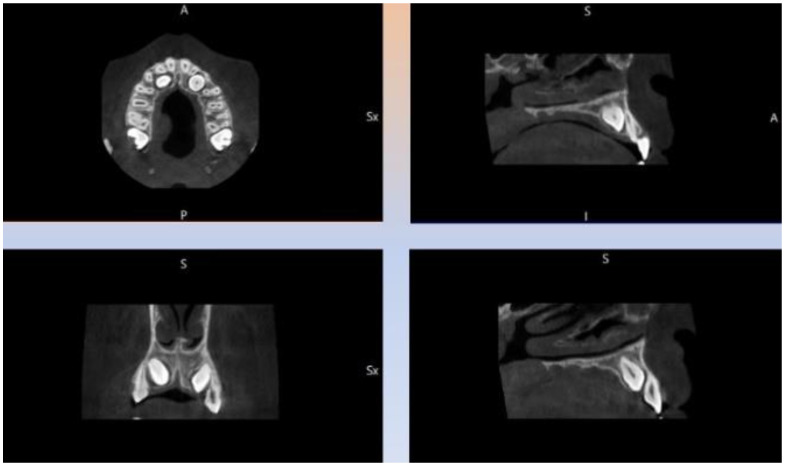
Particular projection of the patient’s CBCT with bilateral inclusion of the maxillary canines.

The study was carried out on a 13 yo patient, referred to the Orthodontics Unit of the Department of Oral and Maxillo-Facial Sciences of “Sapienza” University of Rome. Maxillary primary canines’ persistence in upper arch and absence of the canine bump were observed during the clinical examination. Orthopanoramic X-ray (Figure 1) showed the impaction of both permanent maxillary canines. The patients had no symptoms or discomfort related to dental inclusions. The family history revealed that the patient’s mother had the same orthodontic problem. Cone Beam Computed Tomography (CBCT) (Figure 2) was requested from patient at the beginning of therapy, to accurately evaluate the case before surgery.

The patient was informed with surgical consent about the treatment and potential risks and benefits. An evaluation of the prognosis of impacted canines was performed on OPT by two orthodontists, according to Ericson and Kurol [9]. It was plotted on the opt, major axis of the canine, and perpendicular to the alveolar margin, imagining this as the axis of the canine in its presumed ideal seat. The examination of the CT allowed us to evaluate the three-dimensional morphology of the impacted tooth, its location and inclination in the three planes of space, the depth and type of inclusion, and the relationships with other elements [10].

OPT and CBCT reported an osteo-mucosal deep inclusion of both canines in the palatal site, with an unfavorable prognosis due to their inclination and mesial position, near the central and lateral incisors. In this clinical case, a carbon dioxide laser (CO_2_ laser) was used (SMART US-20D^®^, DEKA, Florence, Italy, 10600 nm) with a power of 4.5 Watts in superpulsed mode (frequency: 80 Hz, fluency: 44.78 J/cm^2^, spot diameter: 400 μm)*;* CO_2_ laser is the most used in surgical procedures involving the soft tissues of the oral cavity, due to the highwater absorption coefficient and to the hemostatic action associated with the thermal effect of the radiation.

It is a gaseous state laser, with superficial action (about 0.2 mm deep), which can be used even in superpulsed mode, to obtain a precise incision in the identified area, completed in shorter time than by other types of lasers [11]. In addition, the high frequency of impulses allows an adequate thermal relaxation of the tissue, with minimal damage to the adjacent healthy areas, due to the spread of heat. Moreover, there is no need to carry out any sutures. In general, a high compliance was described in young patients [12,13], as in the shown case of a 13-years-old patient (Figure 1), suffering from bilateral inclusion of the two upper canines, as shown particular in the CBCT of the patient (Figure 2). Our study was conducted to evaluate the effectiveness of laser surgery as an alternative approach to conventional surgical orthodontic treatment and to evaluate the possible effect of this laser on the canine’s movement after operculectomy.

**Figure 3:**
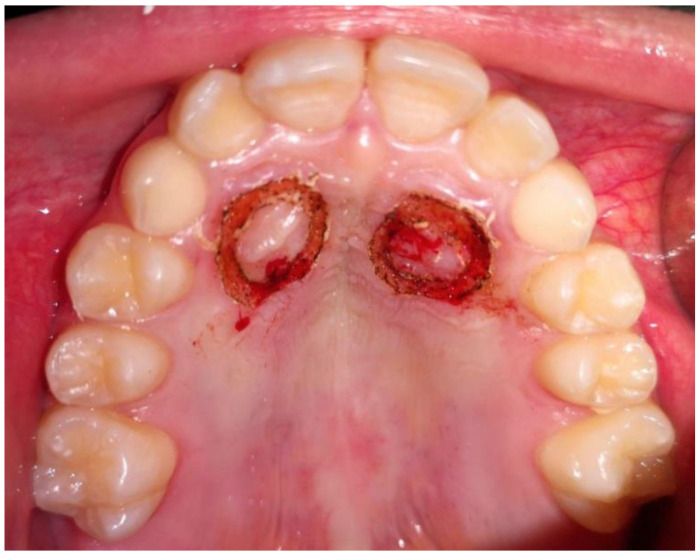
Post-operative photo.

**Figure 4:**
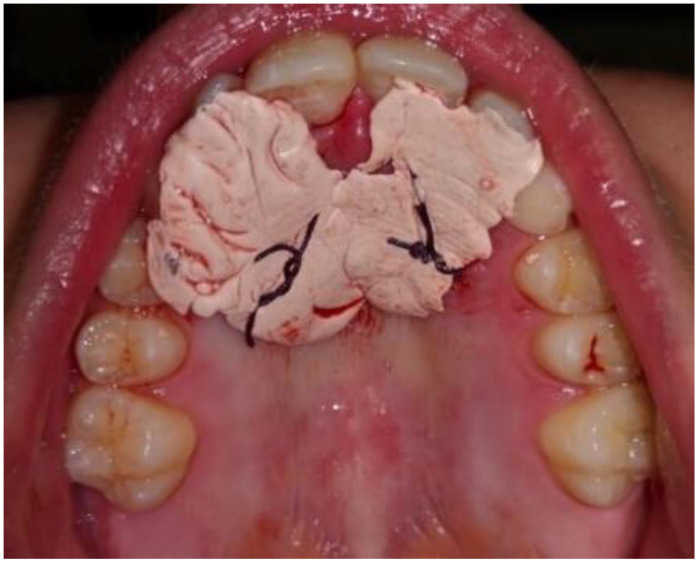
Application of periodontal pack.

**Figure 5:**
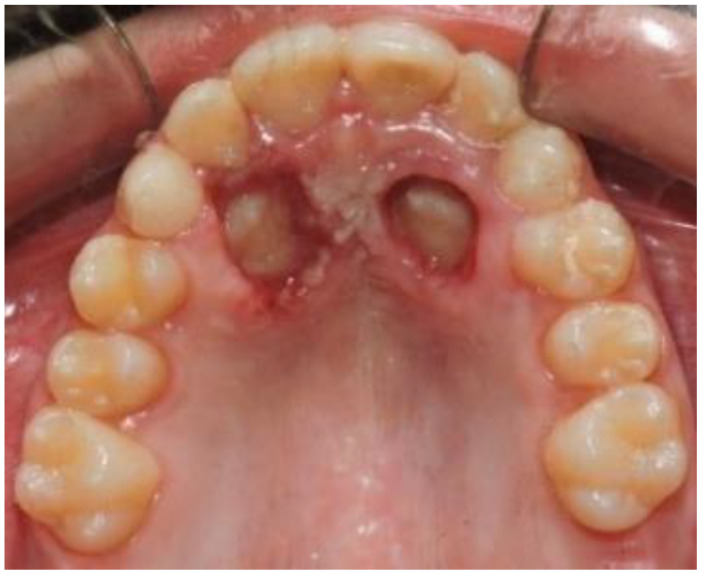
Intra-oral photo 7 days after surgery.

**Figure 6:**
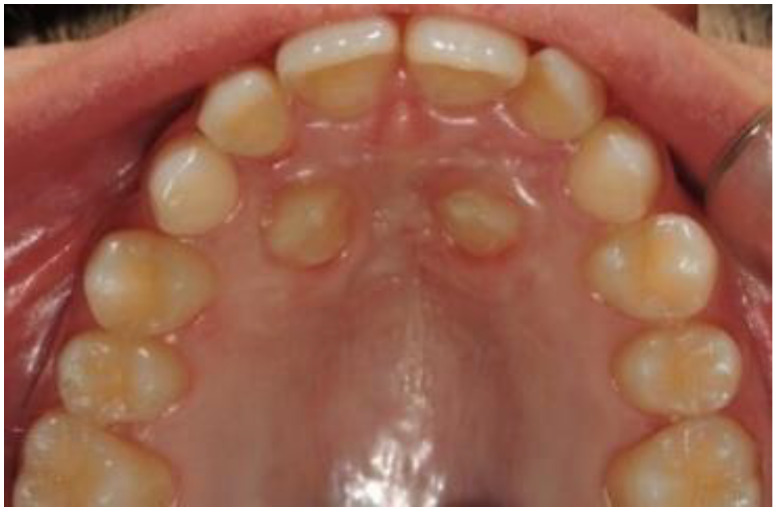
Intra-oral photo 2 months after surgery.

After performing local anesthesia with a 2% solution of mepivacaine with adrenaline 1:100,000, the operculum was performed with CO_2_ laser using the described parameters, whose tip is used not in contact with the tissue surface during the rotary movement necessary to expose the crown of the impacted tooth (Figure 3). Afterwards, since the impacted tooth was covered by bone, an osteotomy was made through a handpiece at low speed and under abundant irrigation with saline, with a rosette bur of ISO 018 diameter (Maillefer^®^, Ballaigues, Switzerland). The drill was used with a tangential sliding movement to the bone tissue, to gradually remove it until the canine’s surface was covered, which must not be damaged. Following the osteotomy, the CO_2_ laser was again applied on the tissue surrounding the canine, to use the possible stimulating effect of laser light on the periodontal ligament, which activates and speeds up tooth movement. Finally, a periodontal compress was applied (Figure 4) (Coe-Pak^®^, GC, Tokyo, Japan), to protect the treated area of the palate for about 7 days, blocked by a suture point of Vicryl 3.0 (Ethicon^®^ V311H 3/0 SH-1 70CM, J&J Medical Devices, Somerville, MA, USA). After surgery, the patient underwent three check-ups: one week (Figure 5) and two months (Figure 6), after laser exposure, to evaluate and monitor, through photographic documentation and digital color scans through the use the intraoral scanner CS3500 (CS3500^®^, Carestream Health, Atlanta, GA, USA), the eruption of the impacted tooth or the possible early closure of the mucosa and lack of appearance of the element in the palate.

One week after the surgery, the Verbal Analog Scale (VAS) was explained and submitted to the patient, who chose a value of 10/100, corresponding to a slight post-operative discomfort and so an absence of pain. During each control visit, a clinical examination was carried out and questions were asked to the patient regarding the occurrence of pain, swelling, bleeding, the need to take drugs, and the reduction of the functionality of the involved area [8]; the answers to the questions about the presence of post-operative problems were all negative. The 3D impressions were then imported into the open-source software MeshLab^®^ (Visual Computing Lab, Pisa, Italy). After importing and superimposing the three scan files at one week, eight and sixteen weeks after surgery, through the “measuring tool”, it was possible to millimetrically measure the distance between the visible cusp of the canine at one week after exposure and the cusp of the canine erupted on the palate at 8 and 16 weeks after laser surgery.

After laser exposure, no orthodontic treatment was begun until the impacted tooth had erupted sufficiently into the palate and the autonomous eruption capacity was noted. When the canines erupted sufficiently into the palate, in a period of four months (16 weeks), a bracket was placed on the tooth, and the upper arch was bandaged with Damon System technique [14,15]. After that, the tooth gradually translated into the dental arch with a metallic ligature and a super elastic CuNiTi arch (0.12).

Extraction of the deciduous canines was avoided until the permanent canines approached sufficiently to the dental arch, in order to improve the aesthetic appearance of the patient during their spontaneous eruption, and to use the canines as space maintainers postponing the use of the orthodontic device.

Therefore, we can state that, at the end of the evaluation period of 4 months, a significant tooth movement was observed. The measurement of the eruption of the canines performed on the superimposed models with MeshLab was 4.74 mm for the right canine and 4.25 mm for the left canine. The speed of eruption was also considered for each canine. The eruption of the canines occurred with a speed of 0.039 mm/day for the right canine and 0.035 mm/day for the left one.

## 3. Discussion and Conclusions

Our surgical approach with laser for the exposure of palatally impacted canines was carried out on the basis of studies present in the literature [16,17] that showed the laser cutting capacity (used at high intensity) for different surgical procedures, such as the removal of soft tissue that surrounds an impacted or partially erupted tooth (opercolectomy), the ablation of gingival proliferation (gingivectomy or gingivoplasty), the excision of maxillary and mandibular frenae (frenectomy) and both incisional and excisional biopsies. Moreover, from other studies [18,19,20], it emerges that low-energy lasers can stimulate the proliferation of osteoclasts, osteoblasts, and fibroblasts, and thereby affect bone remodeling and accelerate tooth movement. Indeed, low-energy laser irradiation enhanced the velocity of tooth movement via RANK/RANKL and macrophage colony-stimulating factor and its receptor expression. The results obtained from these studies have led us to hypothesize the effectiveness of laser in the experimental surgical procedure proposed.

Kokich et al. [21] recommended an alternative disinclusion technique with earlier timing for uncovering palatal canines. They timed the uncovering before the start of orthodontic treatment, the bone over the crown was removed down to the cementum-enamel junction, and an opercolum was made on the palatal mucosa with a surgical scalpel. They supposed that, when the bone and tissue have been removed, these canines would erupt on their own in about 6 to 8 months. This consideration suggests that when the teeth is freed from the overlying tissue, a spontaneous movement of the impacted canine is activated.

Our study was conducted in order to evaluate the effectiveness of laser surgery as an alternative approach to conventional surgical orthodontic treatment and to evaluate the possible effect of this laser on the canine’s movement after the operculectomy. The resolution of this clinical case confirms some data already shown in the late literature [12,22]. The spontaneous eruption times of the canines were shorter than the times described in the literature [20], which could be an advantage attributable to the use of the laser. For sure, the surgical exposure of the maxillary deep impacted canines by CO_2_ laser has shown undoubted advantages [3,8]: absence of bleeding during and after the procedure, no stitches, relative ease and speed of execution, reduced or absent postoperative symptoms, decontaminating action on the treated tissues. Post-operative wound healing was rapid, and patients reported less discomfort in the post-operative period and minor discomfort during speech and chewing. The young patients have resumed normal school and sports activities the day after the surgery. No pain or discomfort was reported in the first post-operative check after a week. We believe that preorthodontic uncovering and autonomous eruption of palatally impacted maxillary canines provides a simplified, predictable, and more esthetic outcome, furthermore, a significant positive factor is that there is no need to carry out the orthodontic traction of the impacted element, which led to less time for orthodontic treatment and, therefore, undoubtedly better compliance by the patient, during the next phase with orthodontic appliance designed to align the ectopic elements in the arch. At the end of the treatment, the patient expressed his positive opinion in general. He claimed that he never had pain, that he was happy to have solved his orthodontic problem in a short time and without special traction devices, but with a spontaneous movement of the teeth, and to have resumed his activities immediately after the surgery.

## Data Availability

The datasets used and/or analyzed during the current study are available from the corresponding author on reasonable request.

## References

[1] Sambataro S., Baccetti T., Franchi L., Antonini F. (2005). Early predictive variables for upper canine impaction as derived from posteroanterior cephalograms. Angle Orthod..

[2] Margot R., Maria C.L., Ali A., Annouschka L., Anna V., Guy W. (2020). Prediction of maxillary canine impaction based on panoramic radiographs. Clin. Exp. Dent. Res..

[3] Impellizzeri A., Palaia G., Horodynski M., Pergolini D., Vernucci R.A., Romeo U., Galluccio G. (2020). CO_2_ laser for surgical exposure of impacted palatally canines. Dent. Cadmos.

[4] Yassaei S., Fekrazad R., Shahraki N. (2013). Effect of Low Level Laser Therapy on Orthodontic Tooth Movement: A Review Article. J. Dent..

[5] Ge M.K., He W.L., Chen J., Wen C., Yin X., Hu Z.A., Liu Z.P., Zou S.J. (2015). Efficacy of low-level laser therapy for accelerating tooth movement during orthodontic treatment: A systematic review and meta-analysis. Lasers Med. Sci..

[6] Sonesson M., de Geer E., Subraian J., Petrén S. (2017). Efficacy of low-level laser therapy in accelerating tooth movement, preventing relapse and managing acute pain during orthodontic treatment in humans: A systematic review. BMC Oral Health.

[7] Palaia G., Impellizzeri A., Tenore G., Caporali F., Visca P., Del Vecchio A., Galluccio G., Polimeni A., Romeo U. (2020). Ex vivo histological analysis of the thermal effects created by a 445-nm diode laser in oral soft tissue biopsy. Clin. Oral Investig..

[8] Impellizzeri A., Di Benedetto S., de Stefano A., Monaco Guercio E., Barbato E., Galluccio G. (2019). General health & psychological distress in children with temporomandibular disorder. Clin. Ter..

[9] Ericson S., Kurol J. (1988). Early treatment of palatally erupting maxillary canines by extraction of the primary canines. Eur. J. Orthod..

[10] Impellizzeri A., Horodynski M., Serritella E., Romeo U., Barbato E., Galluccio G. (2020). Three dimensional evaluation of dental movement in Orthodontics. Dent. Cadmos.

[11] Monteiro L., Delgado M.L., Garcês F., Machado M., Ferreira F., Martins M., Salazar F.J.J. (2019). A histological evaluation of the surgical margins from human oral fibrous-epithelial lesions excised with CO_2_ laser, Diode laser, Er:YAG laser, Nd:YAG laser, electrosurgical scalpel and cold scalpel. Med. Oral Patol. Oral Cir. Bucal..

[12] Testa D., Nunziata M., Mansueto G., Marcuccio G., Motta G. (2019). CO_2_ Laser for the Treatment of Auricle Schwannoma: A Case Report and Review of the Literature. Am. J. Case Rep..

[13] Impellizzeri A., Midulla G., Romeo U., Barbato E., Galluccio G. (2018). Delayed Eruption of Permanent Dentition and Maxillary Contraction in Patients with Cleidocranial Dysplasia: Review and Report of a Family. Int. J. Dent..

[14] Impellizzeri A., Putrino A., Zangrillo C., Barbato E., Galluccio G. (2019). Efficiency of self-ligating vs conventional braces: Systematic review and meta-analysis. Dent. Cadmos.

[15] Putrino A., Impellizzeri A., Pavese L., Barbato E., Galluccio G. (2019). Orthodontic treatment and third molars development: Longitudinal study on radiographs. Dent. Cadmos.

[16] Verma S.K., Maheshwari S., Singh R.K., Chaudhari P.K. (2012). Laser in dentistry: An innovative tool in modern dental practice. Natl. J. Maxillofac. Surg..

[17] Sulieman M. (2005). An overview of the use of lasers in general dental practice: 2. Laser wavelengths, soft and hard tissue clinical applications. Dent. Update.

[18] Fujita S., Yamaguchi M., Utsunomiya T., Yamamoto H., Kasai K. (2008). Low-energy laser stimulates tooth movement velocity via expression of RANK and RANKL. Orthod. Craniofac. Res..

[19] Karu T.I. (2008). Mitochondrial signaling in mammalian cells activated by red and near-IR radiation. Photochem. Photobiol..

[20] Eells J.T., Henry M.M., Summerfelt P., Wong-Riley M.T., Buchmann E.V., Kane M., Whelan N.T., Whelan H.T. (2003). Therapeutic photobiomodulation for methanolinduced retinal toxicity. Proc. Natl. Acad. Sci. USA.

[21] Kokich V.G. (2010). Preorthodontic uncovering and autonomous eruption of palatally impacted maxillary canines. Semin. Orthod..

[22] La Monaca G., Cristalli M.P., Pranno N., Galluccio G., Annibali S., Pippi R. (2019). First and second permanent molars with failed or delayed eruption: Clinical and statistical analyses. Am. J. Orthod. Dentofac. Orthop..

